# Palladium-Functionalized Nanostructured Nickel–Cobalt Oxide as Alternative Catalyst for Hydrogen Sensing Using Pellistors

**DOI:** 10.3390/nano14201619

**Published:** 2024-10-10

**Authors:** Olena Yurchenko, Mike Benkendorf, Patrick Diehle, Katrin Schmitt, Jürgen Wöllenstein

**Affiliations:** 1 Fraunhofer Institute for Physical Measurement Techniques (IPM), 79110 Freiburg, Germanykatrin.schmitt@ipm.fraunhofer.de (K.S.); juergen.woellenstein@ipm.fraunhofer.de (J.W.); 2 Fraunhofer Institute for Microstructure of Materials and Systems (IMWS), 06120 Halle, Germany; 3 Department of Microsystems Engineering (IMTEK), University of Freiburg, 79110 Freiburg, Germany

**Keywords:** metal oxide, nanostructured catalyst, hydrogen sensing, pellistor

## Abstract

To meet today’s requirements, new active catalysts with reduced noble metal content are needed for hydrogen sensing. A palladium-functionalized nanostructured Ni_0.5_Co_2.5_O_4_ catalyst with a total Pd content of 4.2 wt% was synthesized by coprecipitation to obtain catalysts with an advantageous sheet-like morphology and surface defects. Due to the synthesis method and the reducible nature of Ni_0.5_Co_2.5_O_4_ enabling strong metal-metal oxide interactions, the palladium was highly distributed over the metal oxide surface, as determined using scanning transmission electron microscopy and energy-dispersive X-ray investigations. The catalyst tested in planar pellistor sensors showed high sensitivity to hydrogen in the concentration range below the lower flammability limit (LFL). At 400 °C and in dry air, a sensor response of 109 mV/10,000 ppm hydrogen (25% of LFL) was achieved. The sensor signal was 4.6-times higher than the signal of pristine Ni_0.5_Co_2.5_O_4_ (24.6 mV/10,000 ppm). Under humid conditions, the sensor responses were reduced by ~10% for Pd-functionalized Ni_0.5_Co_2.5_O_4_ and by ~27% for Ni_0.5_Co_2.5_O_4_. The different cross-sensitivities of both catalysts to water are attributed to different activation mechanisms of hydrogen. The combination of high sensor sensitivity to hydrogen and high signal stability over time, as well as low cross-sensitivity to humidity, make the catalyst promising for further development steps.

## 1. Introduction

Progressive climate change and the limited resources of fossil fuels make the transition to alternative fuels without carbon content necessary. Hydrogen (H_2_) is considered one of the most promising energy carriers [1,2]. However, the handling of hydrogen is potentially more dangerous than the use of hydrocarbons. The minimum ignition energy for hydrogen (0.017 mJ) is ten times lower than that for methane (0.23 mJ) [1,3], which is why hydrogen has the highest rating of 4 on the flammability scale. Hydrogen forms flammable mixtures with atmospheric oxygen at concentrations between 4%, the so-called lower flammability limit (LFL), and 75%, the so-called upper flammability limit (UFL). The explosion range is between 18% and 59% hydrogen in an oxygen-containing atmosphere [4]. Therefore, fast and reliable measurement of hydrogen, especially at concentrations below the LFL, is crucial to monitor the formation of potentially explosive mixtures in the air and prevent the risk of fire and hydrogen explosions. In gas sensors for safety applications, alarms are typically set at 20% and 40% of the LFL [2]. The detection of hydrogen concentrations down to 1000 ppm is therefore desirable for proper sensor function [5].

Pellistor-type catalytic gas sensors are widely used for the detection of flammable gases, primarily hydrocarbons and hydrogen, in air below their LFL [6,7,8,9]. The principle behind pellistor sensors is to detect the heat released by the oxidation reaction of combustible gases at the catalyst surface. Conventional pellistor sensors consist of two ceramic beads, typically made from porous alumina, covering a fine platinum coil [8,10]. The surface of one bead is coated with a suitable catalyst, which interacts with combustible gas; the other one contains no catalyst and acts as a reference element compensating for external temperature, pressure, and humidity changes. The beads are connected via a Wheatstone bridge circuit to make it easier to compare the coil resistances. The heat generated by the catalytic oxidation of the measured gases causes a change in the resistance of the coil of the active bead due to an increased temperature. As a result, a voltage difference occurs at the output, which is directly related to the concentration of combustible gas.

In order to achieve a high catalytic combustion rate of the combustible gas and thus a better response of the sensor, pellistors are operated at elevated temperatures of well above 300 °C [8], usually at 500 °C [11,12]. Due to their high operating temperatures and their size, conventional pellistors have the disadvantage that they have a high power consumption of between 0.5 and 3.0 W [8]. The power consumption of pellistors can be significantly reduced by replacing conventional bead elements with a planar microheater fabricated on a free-standing or suspended membrane using micro-electromechanical system (MEMS) technology [7,8,11]. However, the gas sensitivity of these planar pellistors is often lower compared to bead-type pellistors due to the smaller size of the active element and the use of a less catalytically active substance [7]. To compensate for this problem, more attention should be paid to the catalyst used.

Palladium (Pd) and platinum (Pt) are used due to their excellent activity in hydrogen oxidation in general and for hydrogen sensing in particular [3,7,8,13,14,15]. Other metals from the platinum group have also been tested as catalysts [6,16]. The low activation energy of hydrogen and oxygen at the surfaces of Pt and especially Pd allows for the dissociation of molecules into chemisorbed atoms already at ambient conditions [5,15,17]. In most cases, noble metals are distributed on a highly porous support material, mostly alumina, which stabilizes the metal catalyst through the support and increases the sensor sensitivity by increasing the catalytically active surface area [6,7,11,14,18]. Porous catalyst layers with increased surface area have also been achieved by using a porous Pt layer, e.g., of colloidal platinum nanoparticles stabilized by monoamine [12] or by using nanostructured Pt or Pd thin films [13,19]. Furthermore, a high noble metal content of up to 40 wt% [7,13,14,19,20] is used in the catalytic layer to further increase the sensor sensitivity and compensate for catalyst degradation.

Although they are often used due to their high activity, the disadvantage remains that noble metals are expensive and rare. Considerable attempts have been made to replace noble metal catalysts with non-noble metal oxides or at least to reduce their quantity while maintaining similar catalytic activity [3]. Inexpensive transition metal oxides such as Co_3_O_4_, MnO_2_, NiO, CuO, CeO_2_, or Fe_2_O_3_ have been the focus of studies on alternative catalysts [8,15,21,22]. As early as 1981, Haruta and Sano found that metal oxides of Co, Mn, Ni, and Cu are active for hydrogen oxidation at intermediate temperatures, and a volcano-like relationship was observed between the catalytic activity and the heat generation of the metal oxides. According to this relationship, Co_3_O_4_ exhibits the highest activity among other non-noble metal oxides [17]. In contrast to alumina and other non-reducible metal oxides like ZrO_2_ or SiO_2_, such metal oxides possess a high oxygen storage capacity and an abundance of oxygen vacancies where oxygen can be adsorbed [8,15]. Metal oxides with a high oxygen storage capacity are better able to provide the lattice oxygen for surface reactions, which ensures high activity in the catalytic processes [8,20,23]. Nonetheless, the non-noble metal oxide exhibits high energy barriers for the activation of molecular hydrogen, especially on perfect metal oxide surfaces [8]. For example, hydrogen dissociation on noble metals like Pt is barrierless (<0.1 eV), whereas on Fe_2_O_3_ surfaces, an activation barrier of 4.4 eV must be overcome [21].

The combination of a reactive metal with a reducible metal oxide has an advantageous effect on the catalytic activity compared to non-reducible alumina and enables a reduction in the metal content in the catalyst. The essential step of hydrogen activation occurs by spillover on noble metals, whereby activated hydrogen atoms migrate from a metal catalyst particle on which they were generated to the catalyst support [15,21,24]. On the support, the hydrogen atoms react with oxygen to form water. The reducibility of the metal oxide is decisive for the hydrogen spillover. Thus, a fast hydrogen spillover is observed on reducible TiO_2_. In contrast, the spillover on alumina is about ten orders of magnitude slower and limited to very short distances to the Pt particle [24].

Co_3_O_4_ has a high reducibility, which is due to its spinel-type structure with variable oxidation states (Co^2+^/Co^3+^) and the lowest binding energy of M–O bonds among all transition metal oxides [1]. The good mobility of the oxygen species in Co_3_O_4_ and its reducibility contribute to its good activity in the catalytic oxidation of hydrogen [15] and even methane [25], which is the most difficult gas to oxidize. The incorporation of Ni heteroatoms into Co_3_O_4_ particles by a coprecipitation reaction leads to the formation of Ni_x_Co_3-x_O_4_ particles with a special sheet-like morphology, which is advantageous for catalysis because it has a high number of structural defects and the reaction sites are better accessible to the reactants [26,27].

In our study, a Pd-functionalized nanostructured spinel nickel–cobalt oxide with the composition Ni_0.5_Co_2.5_O_4_ is presented as a catalyst for hydrogen sensing. The activity of pristine Ni_0.5_Co_2.5_O_4_ for hydrogen sensing was tested. Cobalt was partially replaced by nickel, primarily due to the structure-directing role of nickel atoms in the formation of spinel cobalt oxide particles. Such a structure-directing effect was also observed in the coprecipitation method used [27]. The actual composition of the nickel–cobalt oxide Ni_0.5_Co_2.5_O_4_ was chosen on the basis of earlier work on the stability of Ni_x_Co_3-x_O_4_ [27], since higher nickel contents cause the segregation of nickel oxide.

The sheet-like morphology obtained was well suited for functionalization with palladium. The Ni_0.5_Co_2.5_O_4_ was functionalized with 4.2 wt% (~1.5 at%) Pd during its synthesis by coprecipitation. A higher Pd content in the catalyst could not be achieved with the synthesis route used. Ni_0.5_Co_2.5_O_4_ was not only intended to serve as a carrier for the nanoscale Pd catalyst but also to contribute to the catalytic oxidation reaction due to its superior structural and functional characteristics. For gas tests, the catalyst was deposited by drop-coating onto a micro-hotplate consisting of a Pt heating structure on a thin silicon dioxide membrane. For comparison, the catalyst was characterized with regard to its sensitivity to methane and hydrogen in the concentration range below the LFL of gases.

## 2. Materials and Methods

### 2.1. Materials and Synthesis Procedure

The Pd-functionalized nickel–cobalt oxide (Ni_0.5_Co_2.5_O_4_, with a specific Ni:Co ratio of 1:5) catalyst was synthesized by the precipitation method described in [28]. To obtain the Pd-functionalized Ni_0.5_Co_2.5_O_4_ catalyst, part of the Co precursor was substituted by a Ni precursor and part of the Pd precursor was additionally used in the synthesis. The composition of the final Pd-functionalized Ni_0.5_Co_2.5_O_4_ catalyst was investigated by energy dispersive X-ray (EDX) analysis. The average Ni:Co ratio in Ni_0.5_Co_2.5_O_4_ was the same as that used in the synthesis (1:5). According to EDX measurements, the Pd content in the final Pd-functionalized Ni_0.5_Co_2.5_O_4_ catalyst varied from 4.12 to 4.22 wt%, which was lower than the amount added to the metal salt solution (6 wt% Pd). This is due to the fact that Pd species were removed from the catalyst by washing if they were not firmly anchored to the metal oxide formed. Increasing the Pd concentration in the solution did not lead to a significant increase in the Pd content in the catalyst, which can be attributed to the saturation of the metal oxide surface with Pd.

The Pd-functionalized Ni_0.5_Co_2.5_O_4_ sample was synthesized according to the following precipitation procedure: PdCl_2_ (0.48 g, 6 wt% Pd content) was dissolved in 100 mL deionized water at 22 °C with stirring. Subsequently, Co(NO_3_)_2_ 6H_2_O (14.6 g, >98%; Carl Roth, Karlsruhe, Germany) and Ni(NO_3_)_2_ 6H_2_O (2.91 g, >98%; Carl Roth, Karlsruhe, Germany) were added to the PdCl_2_ solution. The final solution was rinsed in a stream of nitrogen for 15 min. Then, a KOH solution (1 mol L^−1^, 300 mL; Carl Roth, Karlsruhe, Germany) was added with injection of nitrogen gas and constant vigorous stirring for 30 min. The precipitate was collected and washed three times with hot deionized water (60 °C), followed by drying at 130 °C for 24 h. The obtained solid was ground to a powder in a mortar and calcined at 400 °C in air for 24 h, yielding the synthesized catalyst.

The catalyst ink for deposition on the sensor was prepared using a ball mill (PM 100, Retsch, Haan, Germany). For this purpose, the catalyst powder (1.0 g, 35 wt% solid content in final ink) was mixed with 1,2-propylenglycole (1.9 g), which was used as an organic vehicle, and treated in the ball mill for 4 h in a steel jar (12 mL) with 4 mm steel balls.

**Figure 1:**
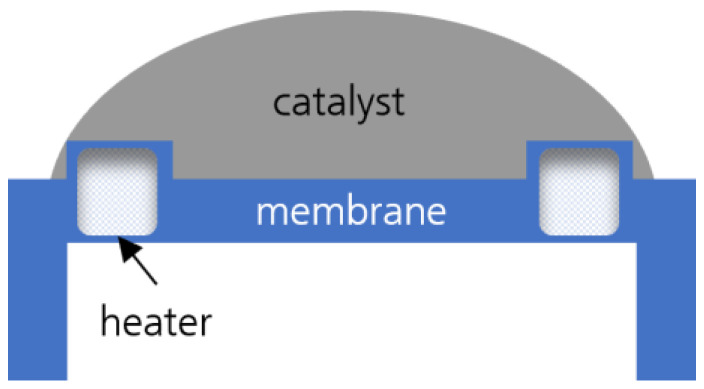
Schematic representation of a sensor substrate. The substrate is coated with the catalyst.

To test the catalyst for hydrogen sensing, MEMS substrates containing a platinum microheater on a suspended membrane were used as sensor substrates (Figure 1). An untreated MEMS substrate was used as a reference sensor element, with the active sensor element of the pellistor additionally covered with a catalyst layer, as shown in Figure 1. Both sensor elements were connected in a Wheatstone bridge circuit. The active and reference sensor elements were located in one branch, while precision resistors with a nominal value of 10 kΩ were arranged in the other branch. To perform the measurements, a heating voltage in the range of 0.8 to 2.6 V was applied to the Wheatstone bridge circuit.

Several sensors were coated with the catalyst ink by drop coating with a pipette and examined (sensors # 1 and 2). The organic vehicle was removed from the sensor by electrical heating. The thickness of the catalyst films on the substrate was in the range of several micrometers.

### 2.2. Characterization Methods

A scanning transmission electron microscope (STEM) (Hitachi HF 5000, Hitachi, Tokyo, Japan) equipped with an in-lens secondary electron (SE) detector and an EDX detector was used to visualize the morphology of the catalyst powder and to analyze the composition of the catalyst. The SE detector provides access to surface information and visualizes the surface topography of the sample. In the following, the images are referred to as SE-STEM, as the images were acquired in STEM mode, although the electrons were not penetrating the sample. To characterize the microstructure of the sample, an HAADF-STEM (high-angular dark field) was employed, which shows mainly material contrast.

The crystalline structure of the catalyst was verified by X-ray diffraction analysis (Empyrean, Malvern Panalytical Ltd., Malvern, UK) using Cu Kα radiation.

The layer morphology of the catalyst on the sensor substrate and the element distribution were investigated by scanning electron microscopy (SEM) using an SU-70 (Hitachi High-Tech Corporation, Tokyo, Japan) at 20 kV voltage.

The gas measurements were carried out at a gas flow of 400 mL min^−1^ with synthetic air, hydrogen, and methane (Air Liquide, Düsseldorf, Germany), whereby the sensors had a constant heating current. The temperature corresponding to the applied heating current was determined by calibration. In order to achieve the operating temperatures of 100 °C, 200 °C, 250 °C, 300 °C, 350 °C, and 400 °C, a constant current of 31 mA, 45 mA, 49 mA, 53 mA, 56 mA, and 59 mA was applied to the substrates. The power consumed was between 24 mW and 155 mW. The resulting sensor response was measured as a voltage difference using a Wheatstone bridge. The measurement chamber had a volume of 150 mL. A humidification device (HovaCal from IAS, Oberursel, Germany) was used to obtain accurate target concentrations of the gases and the required gas humidity. The relative humidity in the chamber was calculated from the given absolute humidity and temperature measured in the chamber simultaneously with the voltage difference. The relative humidity achieved in the chamber was dependent on the ambient temperature.

## 3. Results

### 3.1. Particle Structure and Morphology of the Catalyst

**Figure 2:**
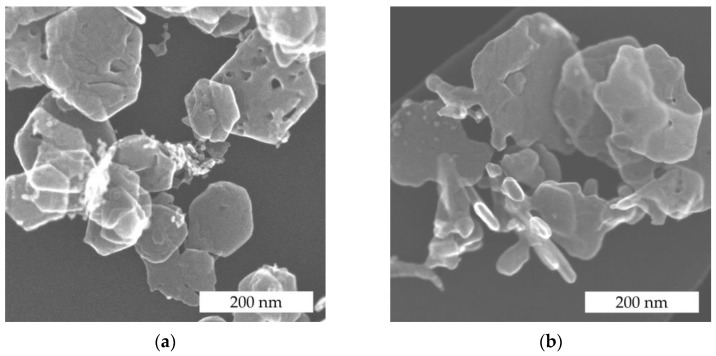
SE-STEM images of (**a**) pure Ni_0.5_Co_2.5_O_4_ and (**b**) Pd-functionalized Ni_0.5_Co_2.5_O_4_. Pd-functionalized Ni_0.5_Co_2.5_O_4_ did not form hexagonal structures and is more fragmented.

Previous work on nickel-modified cobalt oxides Ni_x_Co_3−x_O_4_ (x = 0.5 and 1) prepared by coprecipitation with KOH has shown that the addition of Ni leads to the formation of particles with a specific morphology different from pristine Co_3_O_4_ [27]. However, the cubic Co_3_O_4_ spinel structure was retained for Ni_0.5_Co_2.5_O_4_ (Supplementary Information, Figure S1). Such a nanostructured particle morphology with an increased surface-to-volume ratio is advantageous for carrying noble metal particles and for the fabrication of catalytic layers with high macroporosity. For example, the Ni_0.5_Co_2.5_O_4_ oxide (Figure 2a) with a Ni:Co ratio of 1:5 shows layers with hexagonal morphology that exhibit various structural defects. The surface defects on the nanostructured metal oxide usually lead to improved oxygen adsorption on this surface by changing the gas adsorption properties and thus the reactivity of the surface [5,22]. The Ni_0.5_Co_2.5_O_4_ functionalized with Pd (Figure 2b) retains the sheet-like morphology of the pristine oxide (Figure 2a), but no sheets with hexagonal shapes can be observed. Obviously, the presence of Pd during metal oxide precipitation hinders the formation of hexagonal structures of Ni_0.5_Co_2.5_O_4_. Pd-functionalized Ni_0.5_Co_2.5_O_4_ exhibits a higher number of sheet fragments than the pristine metal oxide.

**Figure 3:**
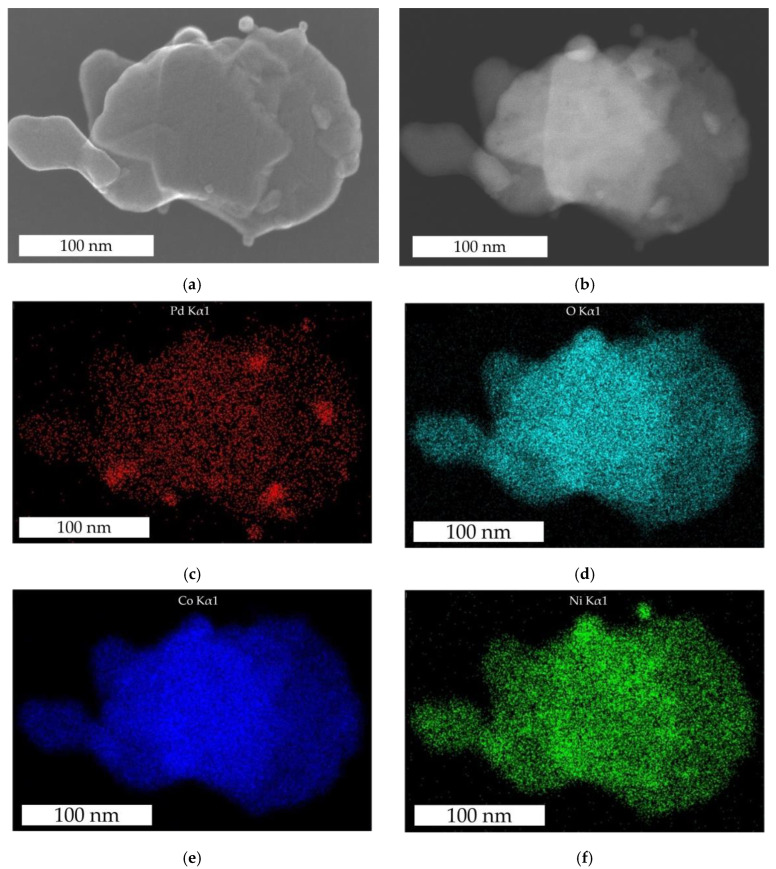
EDX analysis of the Pd-functionalized Ni_0.5_Co_2.5_O_4_ catalyst: SE-STEM (**a**) and HAADF-STEM (**b**) images, and the corresponding element distribution maps of Pd (**c**), O (**d**), Co (**e**), and Ni (**f**).

The element distribution of Pd, O, Co, and Ni elements in the Pd-functionalized Ni_0.5_Co_2.5_O_4_ catalyst is shown in Figure 3. The comparison of SE-STEM and HAADF-STEM images (Figure 3a,b) shows that the layer consists of several fused thin layers and particles. In addition, the sheets show surface defects (see also the Supplementary Information, Figure S2). The distribution of O, Co, and Ni (Figure 3d–f) correlates with each other, indicating a uniform composition of the structure, which is important for the stability of the catalyst. However, one individual Ni nanoparticle is also attached to the sheet. An even higher degree of Ni segregation was observed in NiCo_2_O_4_ with a higher Ni:Co ratio of 1:2 [27]. The uniform distribution of Co and Ni in the structure is important for the thermal stability of the catalyst, especially at higher temperatures (≥450 °C). The thermal stability of catalysts with non-uniform atomic distribution in the structure is lower due to the lower entropy. The pursuit of higher orders and entropy favors thermally activated atomic diffusion and impairs the catalytic stability of such materials. The Pd catalyst (Figure 3c) is present in the form of larger nanoparticles with a particle size up to 15 nm. At the same time, the Pd is distributed over the entire surface of the Ni_0.5_Co_2.5_O_4_ catalyst (see also the Supplementary Information, Figure S3).

Larger Pd nanoparticles are crucial for methane oxidation [23,29,30], while a large number of finely dispersed Pd on the metal oxide surface is beneficial for hydrogen oxidation, as any accumulation of Pd atoms can act as a catalytic site [22] that contributes to hydrogen activation, e.g., through hydrogen spillover. Most catalysts are prepared through impregnation of the metal oxide using metal salts, where Pd particles are simply attached to the surface of the metal oxide [7,10,16,21,31]. The functionalization of metal oxides with Pd and Pt, performed in situ during the formation of metal oxide particles, can initiate the incorporation of metal atoms into the metal oxide structure, as reported by Madras et al. [15]. Madras et al. reported the ionic substitution of Pd and Pt in the Co_3_O_4_-ZrO_2_ composite support observed by PEG-assisted sonochemical synthesis. This was confirmed by distortion in the d-spacing of lattice planes for Pd (111) and Pt (200). The noble metal atoms incorporated into the metal oxide matrix are more strongly bound to the metal oxide host, thus providing a stronger interaction with the metal support, which can lead to improved catalytic performance. The disturbances in the sheet formation of Ni_0.5_Co_2.5_O_4_, surfaces defects, and a high degree of Pd dispersity observed for the Pd-functionalized metal oxide indicate the incorporation of Pd atoms into the oxide structure. Strong metal-oxide interactions between Pd and reducible Ni_0.5_Co_2.5_O_4_ should prevent the aggregation of nanosized Pd during sensor operation [15,21].

In addition, nanosized Pd is preferred for application in catalysts [5]. Nanostructured Pd is less susceptible to mechanical damage caused by hydrogen adsorption, which is particularly pronounced in bulk systems [8]. Chemisorbed redundant H atoms diffuse from the surface of the Pd into the subsurface and then into the bulk. In the bulk, H species form a solid solution with Pd, an α-phase at lower hydrogen pressure, and a β-phase with increasing hydrogen content. Due to a larger lattice constant of the β-phase compared to the Pd host, its formation induces significant lattice expansion, which leads to damage upon repeated hydrogen absorption and desorption [5]. However, the phase transformation under hydrogen exposure is limited in fine Pd nanoparticles due to the limitation of their structure, which has a positive effect on the lifetime of hydrogen sensors based on these noble metals. In addition, reducible supports like TiO_2_ [24] or Ni_0.5_Co_2.5_O_4_ promote the surface migration of activated hydrogen atoms from metal particles to the support itself. This should limit the saturation of hydrogen atoms on the surface of Pd particles.

### 3.2. Morphology of the Catalytic Layer

Porous catalyst layers are commonly prepared by dropping ink onto the microheater [7] or by using a dispensing technique [11,13,19]. In the present work, the catalyst was deposited on the MEMS sensor substrate by dropping the catalyst ink using a pipette, which is a simplified kind of dispensing technique used to rapidly prepare catalytic layers. However, there is some variation in the coated area of individual sensors as the deposition was performed manually.

**Figure 4:**
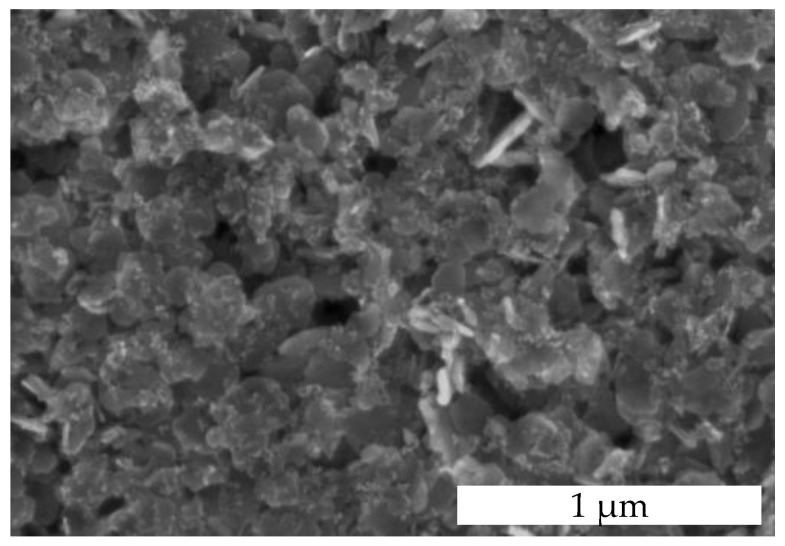
SEM images of the Pd-functionalized Ni_0.5_Co_2.5_O_4_ layer.

The layer morphology of the catalyst is shown in Figure 4. It shows a highly porous layer composed of sheets and small nanoparticles covering the sheets. The layer contains macropores of different sizes that increase the active surface area and facilitate the removal of water molecules from the reaction sites. The larger catalytically active surface area due to layer porosity is crucial for planar pellistors, as they generally have a lower gas sensitivity than bead-type pellistors [7,8,12].

The increased porosity leads to increased catalytic activity. Otherwise, the porosity negatively affects the thermal conductivity of the layer, which plays a significant role in the formation of the sensor signal in catalytic sensors. Thermal conductivity is the most efficient way to conduct the reaction heat from the reaction sites on the surface to the detector. High thermal conductivity is crucial for a fast gas sensor response and a fast recovery time. However, for layers with low thermal conductivity, local hotspots with significantly higher than average temperatures can form at highly active catalytic sites, which in turn leads to non-uniform degradation of the catalyst [17].

The thermal conductivity of dense alumina ceramics is about 17 W m^−1^ K^−1^ at 300 °C, while the corresponding value for highly porous alumina with 46.5% porosity is reduced to 4 W m^−1^ K^−1^ [32]. Mesoporosity also has a negative impact on the thermal conductivity of Co_3_O_4_. Bulk Co_3_O_4_ has a similar thermal conductivity to bulk alumina with a value of 39.3 W m^−1^ K^−1^ at room temperature. In contrast, mesoporous Co_3_O_4_ particles prepared using the KIT-6 template with an extremely high porosity of 45.5% exhibit an exceptionally low thermal conductivity of 0.51 W m^−1^ K^−1^ at room temperature. Therefore, mesoporous Co_3_O_4_ catalysts are not suitable for use in catalytic sensors, as reported by Lyu et al. [33]. Otherwise, the thermal conductivity of Co_3_O_4_ nanoparticles is less affected. For Co_3_O_4_ nanoparticles with an average particle size between 5 nm and 400 nm, the thermal conductivity is 12 to 25 W m^−1^ K^−1^ at 300 K [34]. Consequently, the particle structure is primarily decisive for the thermal conductivity. The nanostructured particles of the Pd-functionalized Ni_0.5_Co_2.5_O_4_ catalyst have a dense structure (Figure 2b and Figure 3) with some surface defects, which should be beneficial for good thermal conductivity. Thus, dense particles of the metal oxide should ensure good thermal conductivity, while the particle arrangement ensures high macroporosity in the layer.

### 3.3. Gas Characterization of the Catalyst

**Figure 5:**
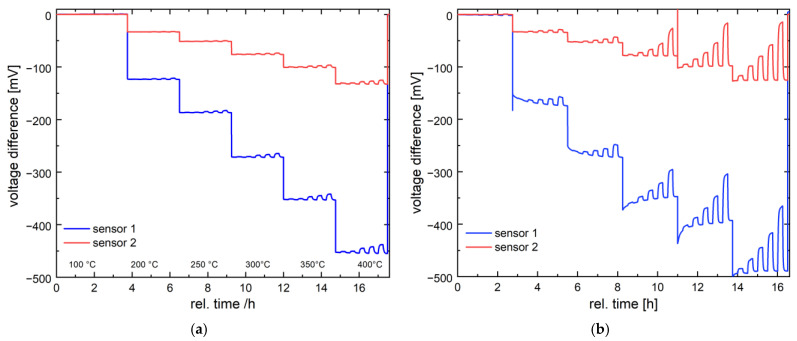
Temperature-dependent dynamic sensor signal of the Pd-functionalized Ni_0.5_Co_2.5_O_4_ catalyst to various concentrations (1000 ppm, 3000 ppm, 5000 ppm, 7000 ppm, and 10,000 ppm) of methane (**a**) and hydrogen (**b**) obtained with sensors 1 and 2 in dry air at 100 °C, 200 °C, 300 °C, 350 °C, and 400 °C.

The Pd-functionalized nanostructured Ni_0.5_Co_2.5_O_4_ catalyst was tested for its response towards methane and hydrogen gas in the concentration range of interest for pellistor applications (2.5–25% of LFL). Previous studies of Ni_0.5_Co_2.5_O_4_ catalyst using differential scanning calorimetry (DSC) [25] demonstrated its catalytic activity towards methane [27]. It was expected that the Pd-functionalized Ni_0.5_Co_2.5_O_4_ catalyst is even more sensitive to methane. To compare the gas sensitivity of the catalyst, methane and hydrogen were tested at six different temperatures (100 °C, 200 °C, 250 °C, 300 °C, 350 °C, and 400 °C) and five gas concentrations (1000 ppm, 3000 ppm, 5000 ppm, 7000 ppm, and 10,000 ppm). Figure 5 shows the dynamic signal of the two sensors. The observed deviation in the signal level of these sensors is due to the different sizes of the catalytic area on the substrate. The sensor response increases for both gases with increasing operating temperature, although the signal amplitude for hydrogen is up to 10 times higher than that for methane at the respective temperature. For hydrogen, a significant signal is obtained for all tested concentrations already at 200 °C, while for methane, depending on the sensor used, all concentrations can only be detected from 300 °C or 350 °C. The significantly higher signal for hydrogen [35] is primarily due to its high reactivity and high oxidation rate due to the low energy activation barrier. In addition, the Pd-functionalized nanostructured Ni_0.5_Co_2.5_O_4_ catalyst is modulated with finely distributed Pd particles to enhance hydrogen spillover. In contrast, methane is the least reactive gas in oxidation reactions. To achieve high intrinsic catalytic activity for methane oxidation, catalysts containing a high number of noble particles of a certain size are required [29]. The Pd-functionalized Ni_0.5_Co_2.5_O_4_ catalyst with a rather insufficient amount of larger Pd particles but with a high amount of fine Pd particles is less suitable for methane oxidation. Due to the different sensor sensitivities to methane and hydrogen, the two gases can be easily distinguished.

**Figure 6:**
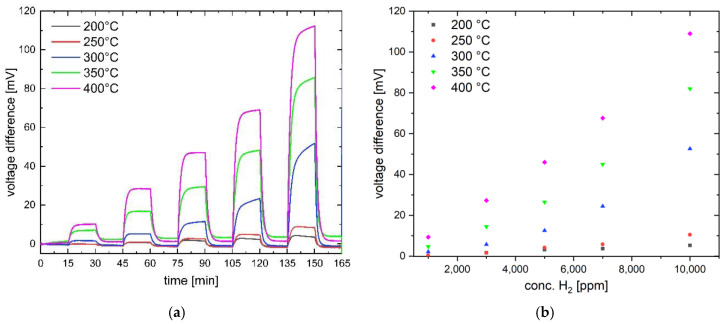
Temperature dependence of the dynamic signal of sensor 2 (Pd-functionalized Ni_0.5_Co_2.5_O_4_ catalyst) measured at five hydrogen concentrations (1000 ppm, 3000 ppm, 5000 ppm, 7000 ppm, 10,000 ppm) (**a**) and the corresponding sensor response (**b**).

Figure 6 shows the signal from gas sensor 2 at different hydrogen concentrations as a function of the operating temperature. Sensor 1 has a higher sensitivity. After hydrogen exposure (Figure 6a), the signal recovers to the baseline, indicating the reversibility of the catalytic process. However, the sensor does not yet show perfect linearity of the concentration-dependent reaction, especially at higher hydrogen concentrations. The deviation from linearity is due to the. measurement method used, where sensors were operated with the current source without temperature compensation. In this case, the temperature at the catalyst surface at high hydrogen concentrations is higher than the specified operating temperature, which in turn leads to a nonlinear increase in the reaction heat due to a reaction acceleration. To avoid this effect, the Wheatstone bridge must be operated with a voltage source instead of a current source.

**Figure 7:**
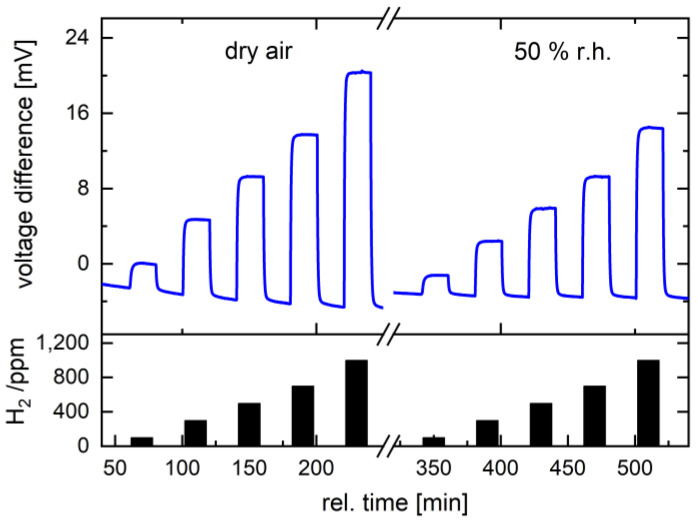
Hydrogen concentration-dependent dynamic sensor signal (blue line) of pristine Ni_0.5_Co_2.5_O_4_ measured in dry and humid air (50% RH) at 400 °C and 1000 ppm, 3000 ppm, 5000 ppm, 7000 ppm, and 10,000 ppm hydrogen.

Overall, the sensors based on the Pd-functionalized Ni_0.5_Co_2.5_O_4_ catalyst with 4.2 wt% Pd content show superior hydrogen sensitivity (e.g., 109 mV/10,000 ppm hydrogen at 400 °C for sensor 2). For comparison, the gas sensor based on pristine Ni_0.5_Co_2.5_O_4_ oxide without Pd functionalization has achieved a sensitivity of 24.6 mV/10,000 ppm hydrogen at 400 °C in dry air (Figure 7). Figure 7 shows the sensor signal of pristine Ni_0.5_Co_2.5_O_4_ oxide measured in dry and humid air at 50% r.H. The 4.6-fold increase in the sensor signal for Pd-functionalized Ni_0.5_Co_2.5_O_4_ in dry air clearly shows that Pd plays a crucial role in hydrogen oxidation. The sensitivity achieved with a particular Pd-functionalized catalyst at 400 °C is comparable to the sensitivity of the microheater-based catalytic sensor reported by Napolskii [7]. The sensor based on porous alumina impregnated with a 3PdPt catalyst with a total metal content of 13.8 wt% showed a sensitivity of 76 mV/10,000 ppm hydrogen in dry air at 500 °C [7]. Other catalytic sensors show significantly lower hydrogen responses [14,16,36], although a direct comparison of sensor amplitudes is difficult due to differences in the sensor substrates used.

Humidity is an important factor that can significantly affect the sensor response by deactivating the active hydrogen adsorption sites through blockage by water molecules. Several studies mentioned a reduced response of catalytic hydrogen sensors based on Pd catalysts under humid conditions [5,14]. For example, a 25–30% reduction in the response due to humidity and an even greater inhibition at low operating temperatures was observed [14]. However, other authors reported that humidity has little or no effect on the sensor response [6,7,10,34]. Ultimately, the main difference in the effect of humidity on the sensor response of noble catalysts seems to be the operating temperature. In contrast to low temperatures (≤300 °C), at high operating temperatures (≥500 °C), the adsorption of water molecules on the surfaces is limited, and therefore, no influence of humidity is observed.

**Figure 8:**
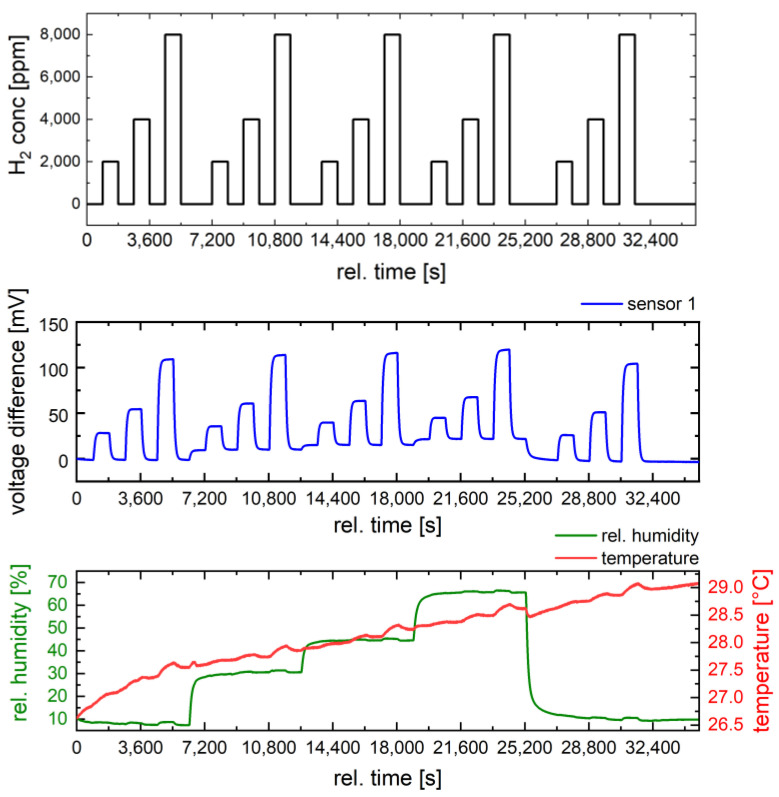
Effect of humidity (green line) on the sensor signal (blue line) of the Pd-functionalized Ni_0.5_Co_2.5_O_4_ catalyst for three different hydrogen concentrations (black line) measured at 350 °C. Temperature variation during the measurements in the chamber is depicted as a red line.

Next, the influence of humidity on the sensor response was investigated at 400 °C for three hydrogen concentrations (2000 ppm, 4000 ppm, and 8000 ppm) (Figure 8). For our investigations, a moderate operation temperature of 400 °C was chosen to reduce the susceptibility of the sensor to humidity and to limit power consumption. The humidity level was varied from 10% RH to 65% RH, while the ambient temperature in the chamber was controlled during measurements.

**Figure 9:**
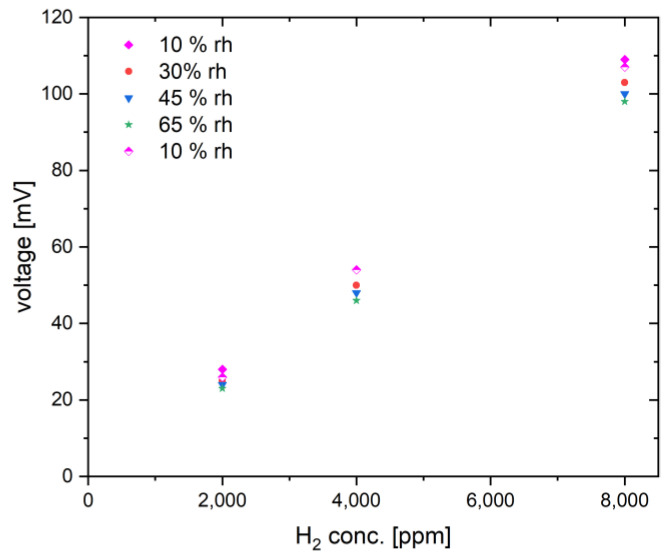
Concentration dependence of the measured sensor signal of the Pd-functionalized Ni_0.5_Co_2.5_O_4_ catalyst at four humidity levels measured at 350 °C.

**Figure 10:**
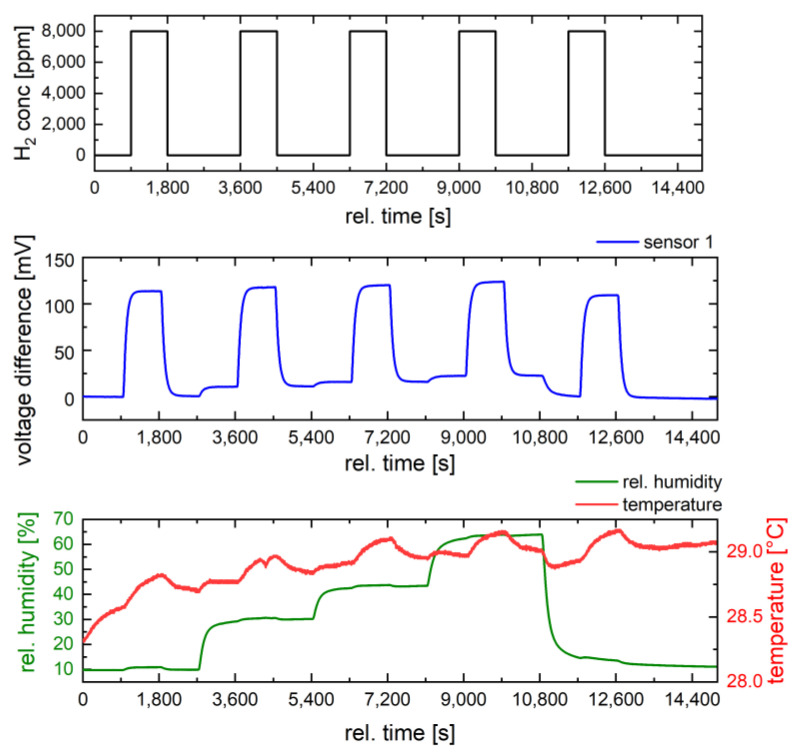
Stability of the sensor signal (blue line) of the Pd-functionalized Ni_0.5_Co_2.5_O_4_ catalyst investigated for 8000 ppm hydrogen (black line) under humidity variation (green line) from 10% to 65%. Temperature variation during the measurements in the chamber is depicted in red.

First, the influence of humidity on the sensor signal can be seen in the baseline shift. Changing the relative humidity from 0% to 65% causes a baseline shift comparable to a sensor response to 2000 ppm hydrogen in air. Figure 9 shows the sensor response to different humidity levels. It can be seen that the sensors are sensitive to humidity variations. The reduction in sensor response with increasing humidity increases with hydrogen concentration. At 2000 ppm hydrogen, the response decreases from 28 mV at 10% RH to 23 mV at 65% RH, while at 8000 ppm hydrogen, it decreases from 109 mV to 98 mV. The value of 11 mV corresponds to a hydrogen concentration of about 800 ppm (2% of LFL) and a determination error of 10%, which is an acceptable measurement error. However, for an application that requires high accuracy in concentration determination, humidity should also be measured and compensated. It is noticeable that pristine Ni_0.5_Co_2.5_O_4_ oxide shows a much stronger dependence of the sensor signal on humidity (Figure 7). Compared to dry air, the sensor signal is reduced by up to 30% at 50% RH. At 10,000 ppm hydrogen, the signal is reduced by 6.7 mV from 24.6 mV to 17.9 mV, which corresponds to a hydrogen concentration of 2455 ppm or almost 6% of the LFL. The reason for the different cross-sensitivities of Pd-functionalized and pure metal oxide towards water is probably the different activation mechanisms of hydrogen and oxygen. In the pure metal oxide, the activation of hydrogen and oxygen occurs exclusively on the metal oxide itself by chemisorption, while Pd contributes the additional activation sites, especially for hydrogen spillover, in the Pd-functionalized Ni_0.5_Co_2.5_O_4_ oxide [15]. It is obvious that the Pd active sites are less affected by water than the Ni_0.5_Co_2.5_O_4_ oxide active sites. Hydrogen activation is likely mainly hindered on the Ni_0.5_Co_2.5_O_4_ catalyst under humid conditions, since such a pronounced inhibitory effect of water was not observed in methane measurement on NiCo_2_O_4_ [26]. Finally, the stability of the sensor signal for a single concentration of 8000 ppm hydrogen (20% of LFL) was investigated under humidity variations, as shown in Figure 10. An additional cyclic stability test is provided in the Supplementary Information (Figure S4). The sensor shows the same signals as in Figure 8. In addition to a baseline shift with humidity variations and a slight decrease in the sensor response with increasing humidity, a high baseline stability and high reproducibility of the response (100 mV/8000 ppm hydrogen at 65% RH) are observed. The high catalyst stability under the operating conditions of the pellistor (sensor 1) was observed. Sensor 1 was subjected to further gas tests (e.g., investigating the stability of the sensor response at 8000 ppm hydrogen at different humidity levels), without any effect on the sensor response being observed. Such a high signal stability confirms the assumption that the nano-sized Pd catalyst is well stabilized by strong metal-metal oxide interactions on the Ni_0.5_Co_2.5_O_4_ oxide between Pd and the reducible Ni_0.5_Co_2.5_O_4_ oxide. However, after repeating the gas tests at higher hydrogen concentrations with very high gas response up to 600 mV, the catalyst layer detached from the sensor substrates. Therefore, the adhesion of the catalyst layer to the MEMS substrate is a problem which should be improved upon in further work by optimizing the ink formulation.

In summary, Pd-functionalized Ni_0.5_Co_2.5_O_4_ (4.2 wt% Pd content) synthesized by coprecipitation with KOH was investigated as a catalyst in a pellistor for hydrogen sensing. The catalyst showed a preferential microstructure and morphology with a highly dispersed metal catalyst on a reducible metal oxide support with intrinsic catalytic activity. The highly dispersed nanosized Pd provided a high number of active sites for hydrogen spillover, while the reducible Ni_0.5_Co_2.5_O_4_ supported hydrogen oxidation by providing oxygen species for reactions due to its high oxygen storage capacity. Such positive effects were reflected in a high sensor response to hydrogen, which was 4.6 times higher than the response of pristine Ni_0.5_Co_2.5_O_4_ at 400 °C. Furthermore, unlike the Ni_0.5_Co_2.5_O_4_ support, the response of Pd-functionalized Ni_0.5_Co_2.5_O_4_ was hardly affected by humidity in the range between 10% and 65% RH (29.0 °C). This is in contrast to Ni_0.5_Co_2.5_O_4_, which showed a significantly reduced sensor signal at 400 °C and 50% RH. The difference in the cross-sensitivity of the two catalysts to humidity was attributed to the lower inhibitory effect of water on the hydrogen activation occurring on Pd by spillover compared to the hydrogen activation occurring on Ni_0.5_Co_2.5_O_4_ by chemosorption. The other observed advantage of Pd-functionalized Ni_0.5_Co_2.5_O_4_ is that the strong interactions between nanosized Pd and Ni_0.5_Co_2.5_O_4_ enabled high stability of the sensor response in our tests.

The synthesis method used is scalable for industrial applications. Future work will focus on optimizing the catalyst ink to achieve better adhesion of the catalyst layer to the substrate and more uniform film deposition, as well as long-term investigations. It will also be interesting to test the sensor at two operating temperatures (350 °C and 400 °C) to investigate the temperature effect on catalyst aging.

## Data Availability

The original contributions presented in the study are included in the article and Supplementary Materials, further inquiries can be directed to the corresponding author.

## Supplementary Materials

The following supporting information can be downloaded at https://www.mdpi.com/article/10.3390/nano14201619/s1, Figure S1: XRD patterns of Ni_0.5_Co_2.5_O_4_; the reference data are listed below; Figure S2: SE-STEM (a) and HAADF-STEM (b) images of the Pd-functionalized Ni_0.5_Co_2.5_O_4_ catalyst; Figure S3: EDX analysis of the Pd-functionalized Ni_0.5_Co_2.5_O_4_ catalyst: SE-STEM (a) and HAADF-STEM (b) images; the corresponding elemental distribution maps of Pd (c), O (d), Co (e), and Ni (f); Figure S4: Stability of the sensor signal (blue line) of the Pd-functionalized Ni_0.5_Co_2.5_O_4_ catalyst investigated for 8000 ppm hydrogen under humidity variation (green line) from 10% to 65%. Temperature variation during the measurements in the chamber is depicted in red.
