# Supplementary material for: Palladium-Functionalized Nanostructured Nickel–Cobalt Oxide as Alternative Catalyst for Hydrogen Sensing Using Pellistors
Source: Nanomaterials (Basel). 2024 Oct 10;14(20):1619. doi: 10.3390/nano14201619 (PMC11510470; doi:10.3390/nano14201619)
Supplement: Supplementary file 1 [file nanomaterials-14-01619-s001.zip › nanomaterials-3190254-supplementary.pdf]

Supporting Information

Palladium-functionalized nanostructured nickel-cobalt oxide as alternative catalyst for hydrogen sensing using pellistors

Olena Yurchenko[1]*, Mike Benkendorf[1], Patrick Diehle[2], Katrin Schmitt[1,3] and Jürgen Wöllenstein[1,3]

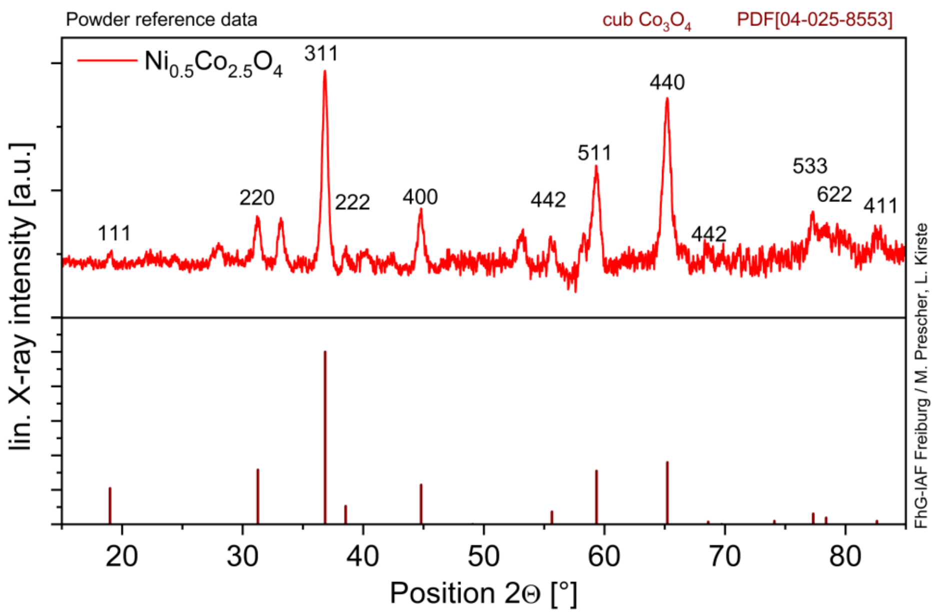

Figure S1. XRD patterns of $Ni_{0.5}Co_{2.5}O_4$; the reference data are listed below.

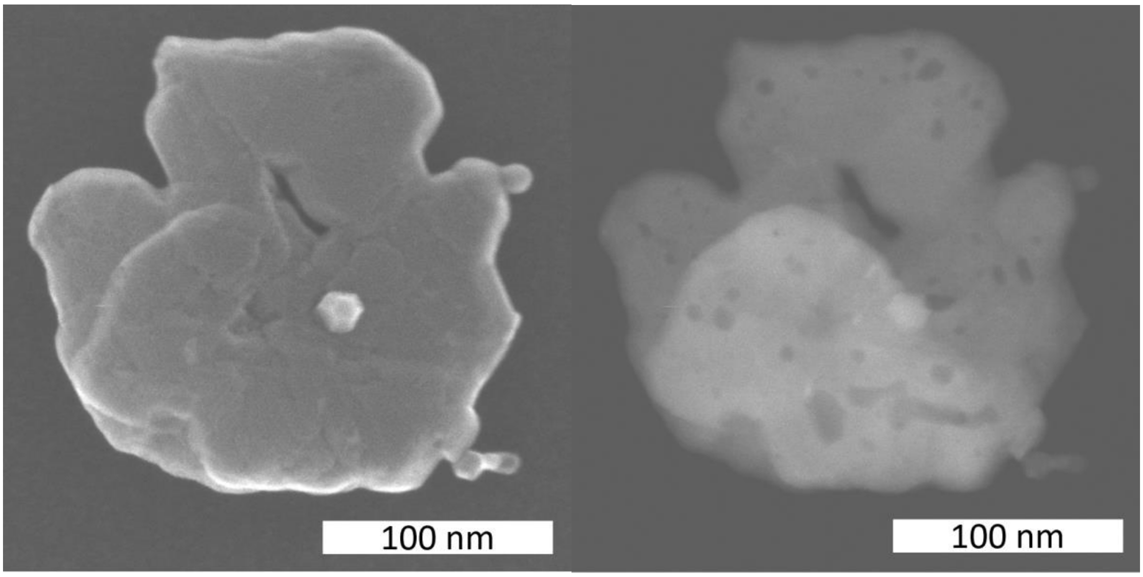

Figure S2. SE-STEM (**a**) and HAADF-STEM (**b**) images of the Pd-functionalized $Ni_{0.5}Co_{2.5}O_4$ catalyst.

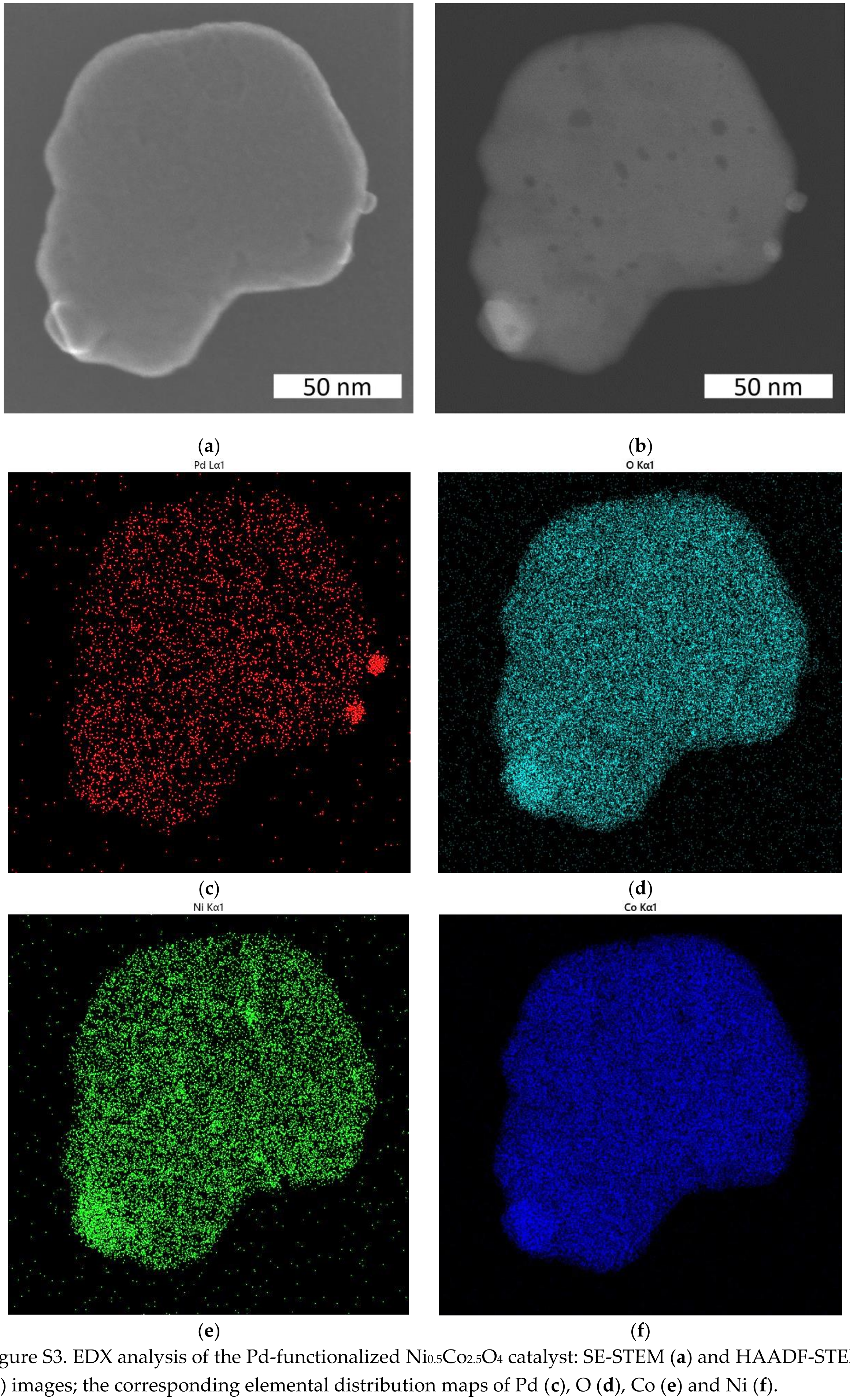

Figure S3. EDX analysis of the Pd-functionalized $Ni_{0.5}Co_{2.5}O_4$ catalyst: SE-STEM (**a**) and HAADF-STEM (**b**) images; the corresponding elemental distribution maps of Pd (**c**), O (**d**), Co (**e**) and Ni (**f**).

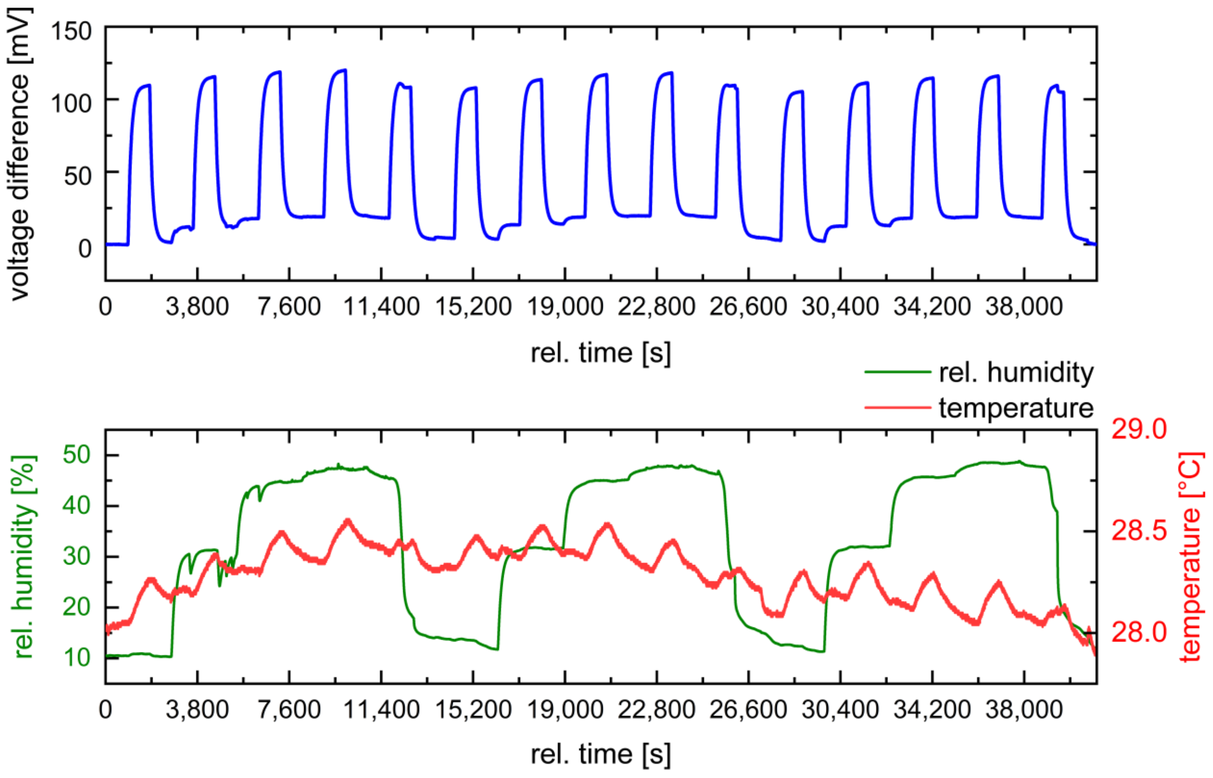

Figure S4. Stability of the sensor signal (blue line) investigated for 8,000 ppm hydrogen under humidity variation (green line) from 10% to 65%. Temperature variation during the measurements in the chamber is depicted in red.
